# Slipper Limpet (*Crepidula fornicata)* Shells Support In Vitro Osteogenesis of Human Adipose-Derived Stem Cells

**DOI:** 10.3390/md21040248

**Published:** 2023-04-17

**Authors:** Arianna De Mori, Umoru Junior Alasa, Alex Mühlhölzl, Gordon Blunn

**Affiliations:** 1 School of Pharmacy and Biomedical Science, University of Portsmouth, St. Michael’s Building, White Swan Road, Portsmouth PO1 2DT, UK; 2 Mikota Ltd., Pembroke Dock, Pembrokeshire, Wales SA72 6AE, UK

**Keywords:** *Crepidula fornicata*, slipper limpet shells, mantle, calcium carbonate, mesenchymal stem cells, osteogenesis

## Abstract

This study aimed to investigate a cost-effective alternative to man-made calcium phosphate ceramics for treating bone defects. The slipper limpet is an invasive species in European coastal waters, and its shells composed of calcium carbonate could potentially be a cost-effective source of bone graft substitutes. This research analyzed the mantle of the slipper limpet (*Crepidula fornicata)* shells to enhance in vitro bone formation. Discs machined from the mantle of *C. fornicata* were analyzed using scanning electron microscopy with energy dispersive spectroscopy (SEM-EDS), X-ray crystallography (XRD), Fourier-transform infrared spectroscopy (FT-IR) and profilometry. Calcium release and bioactivity were also studied. Cell attachment, proliferation, and osteoblastic differentiation (RT-qPCR and alkaline phosphatase activity) were measured in human adipose-derived stem cells grown on the mantle surface. The mantle material was mainly composed of aragonite and showed a sustained Ca^2+^ release at physiological pH. In addition, apatite formation was observed in simulated body fluid after three weeks, and the materials supported osteoblastic differentiation. Overall, our findings suggest the mantle of *C. fornicata* shows potential as a material for fabricating bone graft substitutes and structural biomaterials for bone regeneration.

## 1. Introduction

Trauma, cancer, and congenital disorders result in bone lesions and defects [1]. Consequently, several million people worldwide undergo bone grafting yearly, which has a high economic impact on health systems [2]. There are three main types of bone grafts: autografts, allografts, and xenografts; however, they all possess limitations (i.e., donor site morbidity, immunogenicity, biological variability, low availability), which affect their use. Due to these limitations, alternative man-made and natural bone substitute materials are used clinically for bone regeneration [3]. Calcium carbonate, calcium sulfate, hydroxyapatite and tricalcium phosphate have all been used as bone graft substitutes [4]. Porous hydroxyapatite is one of the most frequently used materials and has been shown to promote in vivo osteoblastic differentiation whilst being osteogenic and osteoconductive [5,6]. This material is mildly osteoinductive. However, this is usually associated with using growth factors such as bone morphogenetic proteins. Hydroxyapatite bone graft substitutes have a low biodegradation rate, which is not always compatible with the growth of new bone [7]. As bone formation progresses on the surface of the graft, the ability of a material to degrade and be replaced by bone tissue is important. Tailored resorption of synthetic bone graft material can induce and improve the capacity of bone required for the repair of fractures by enabling the material to be assimilated with the surrounding living bone tissue. The discovery in 1931 of perfectly Osseo integrated teeth made of nacre (the inner layer of mollusc shells) in Mayan jaw bones encouraged researchers to explore the osteogenic and osteoinductive potential of the mother of a pearl [8]. Marine bivalve shells have a nacre lining that comprises anhydrous calcium carbonate plates cemented by a thin organic matrix (1–5% wt.) [9]. This material has the potential to be used as a bone graft substitute. 

Anhydrous CaCO_3_ exists in three crystalline polymorphs: calcite, aragonite and valerite. Calcite is the most stable and least soluble in water and is found in a trigonal crystalline state in nature. Valerite, on the other hand, is the least stable, exists in a hexagonal crystalline form, and is present in bird eggs and many marine living organisms, such as gastropods and mollusc pearls [10]. Aragonite occurs in the orthorhombic form, commonly found in seashells (e.g., bivalves) and corals. Aragonite is extraordinary biocompatible [11], is denser than calcite, and can be integrated and replaced by bone [12]. The biogenic aragonite in the nacre makes the shells strong enough to protect the shellfish from harsh environments, and the nacre has the potential to be used as a biomaterial with outstanding mechanical properties. The fracture toughness of nacre is 3000 times greater than that of pure aragonite [13], and its mechanical properties are comparable to titanium [14]. Several published articles have suggested that the bone response to nacre combined with its strength makes it a promising candidate for dental implants, whilst nacre powder has been used to reconstruct maxillary defects [8,15,16].

Furthermore, calcium carbonate is more soluble than hydroxyapatite, with a faster resorption rate. Finally, biogenic CaCO_3,_ derived from waste shells or invasive species such as the slipper limpet, would reduce environmental impact and be more economical than a bone graft substitute fabricated from chemicals obtained from limestone [10,17]. Although extensive research has been conducted on the bioactivity, biocompatibility, and osteoinductive properties of nacre from various species such as *Hyriopsis cumingii*, *Arctica islandica*, *Pinctada Maxima*, *Pinctada margaritifera*, *Limnoperna fortunei*, *Anadara granosa*, and pure aragonite from cuttlefish skeletons (*Sepia officinalis*) and corals (*Porites* sp.) [18,19,20,21,22], shells of *C. fornicata* have yet to be studied as a potential biomaterial for bone regeneration.

The slipper limpet, or *C. fornicata*, is a marine gastropod native to eastern North America but invasive to UK and EU waters. It has displaced native species and grown to harmful numbers. Not being a commercial species, *C. fornicata* has no commercial value. However, it is regularly caught as a by-catch of oyster and scallop dredging, often illegally dumped above high tide to naturally deteriorate. In the UK, it is illegal to return slipper limpets to the water or seabed [23]. This invasive species could offer a novel, cost-effective material for bone graft substitutes, incentivizing activities for cleaning up this invasive species whilst providing environmental improvements [16]. These opportunities, together with the increasing demand for large volumes of inexpensive biomaterials from natural resources for the repair of bone defects [24], have led us to investigate the osteogenic potential of this new source of aragonite to produce bone graft substitutes and structural biomaterials made from the mantle of *C. fornicata*. 

This study evaluated, for the first time, the ability of the mantle of *C. fornicata* to enhance the proliferation and differentiation of human adipose-derived stem cells into osteoblasts (OBs). 

## 2. Results

### 2.1. Characterisation of C. fornicata Shells

**Figure 1:**
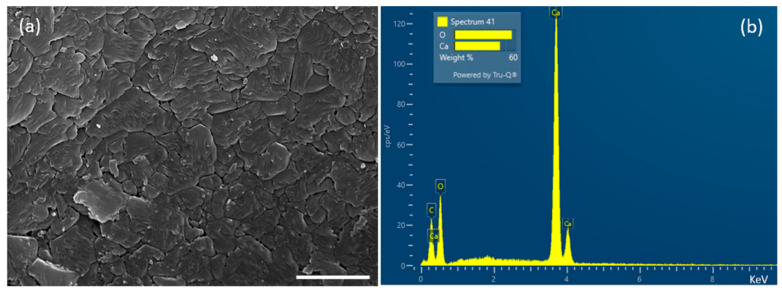
(**a**) SEM image of *C. fornicata* outside surface (scale bar 2 μm), showing interlocking plates. (**b**) EDS compositional analysis of limpet shells, showing the elemental composition of *C. fornicata* shells.

The physicochemical characterization of *Crepidula fornicata* powder (9.07 ± 52.23 µm) was studied by scanning electron microscopy (SEM) combined with energy dispersive spectroscopy (EDS), X-ray diffraction (SEM-EDS, XRD) and Fourier-transform infrared spectroscopy (FT-IR). In the SEM, the surface of *Crepidula fornicata* shells was composed of stacked and interlocking plates and appeared rough (Figure 1a). Although it is impossible to quantify the actual carbon levels as the samples were coated with a thin layer of carbon, the elemental composition from the EDS suggests that calcium and oxygen were both present. Furthermore, the height of the carbon peak suggests that this was also present in the shell (Figure 1b).

**Figure 2:**
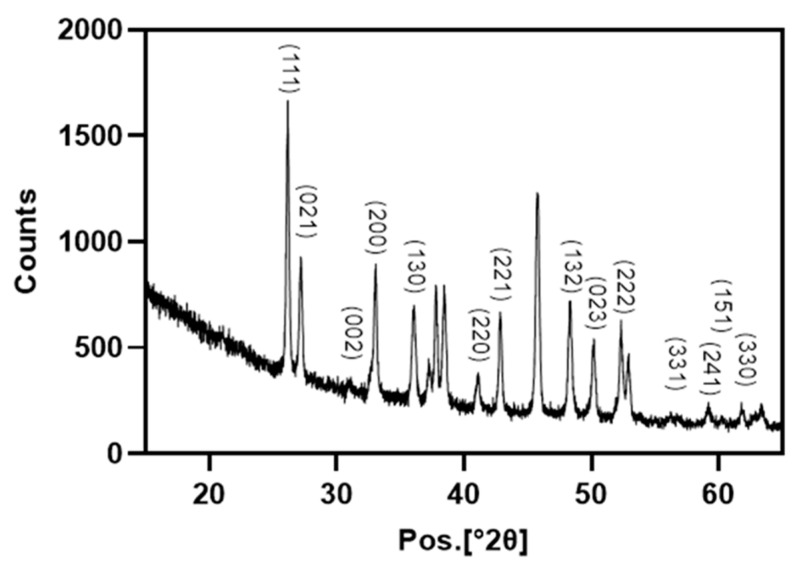
X-ray diffraction patterns of *Crepidula fornicata*’s powder, showing the presence of aragonite.

From X-ray plots, the characteristic peaks identified at angles of diffraction at their respective two theta positions are shown in Figure 2. The X-ray pattern of powdered shells revealed that the main crystalline organization was the orthorhombic structure of calcium carbonate indicative of aragonite. The sharp peaks confirmed the high crystallinity of aragonite-CaCO_3_ (Ref. Code 01-075-9985).

**Figure 3:**
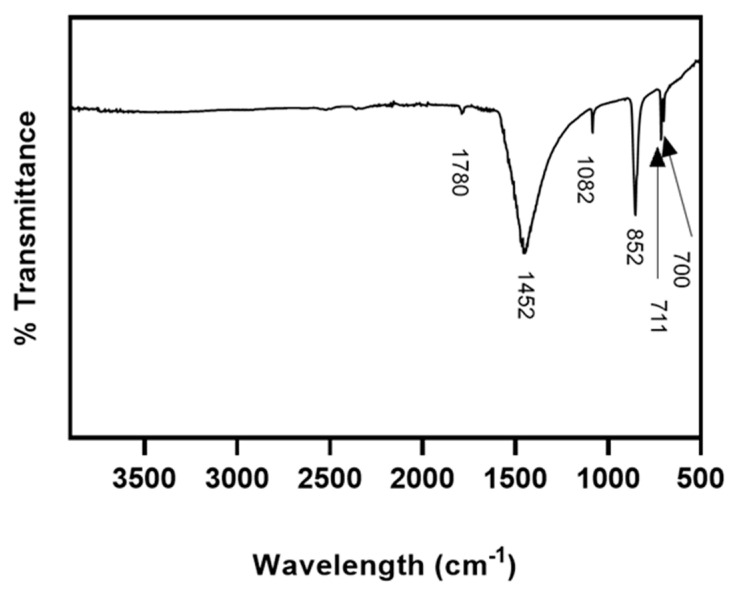
FT-IR spectrum of *C. fornicata* powder, containing aragonite-CaCO_3_.

Fourier-transform infrared spectroscopic analysis (FT-IR) of *C. fornicata* shells confirmed that they were mainly composed of aragonite (Figure 3). The five prominent peaks of aragonite were: a doublet at 711 and 700 cm^−1^ (calcite would have just a single peak) (due to the C-O in-plane bending), 852 cm^−1^ (out-of-plane bending), a weak peak at 1082 cm^−1^ (symmetrical stretching), the strongest peak at ca. 1452 (asymmetrical stretching) and a weak peak, at 1780 cm^−1^ (combination of v1 and v4). No peak was found for phosphate (PO_4_^3^).

### 2.2. Calcium Release Studies from C. fornicata Discs

**Figure 4:**
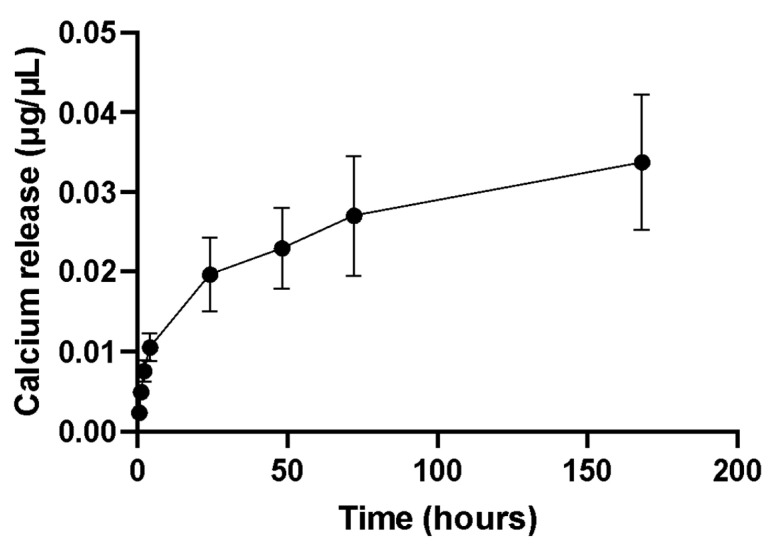
Cumulative calcium release profile from *C. fornicata’s* discs in PBS, at 37 °C, over a period of 7 days. Each data point represents the mean ± SD (*n* = 3).

Figure 4 shows the cumulative Ca^2+^ release in phosphate-buffered saline (PBS) from *C. fornicata* discs (pH 7.4), with a surface area of ca. 70.7 mm^2^, over a period of 7 days. The rate of calcium release was initially fast, but this rate reduced to a sustained release over time. The calcium release profile shows that shell discs from the mantle are not chemically inert materials, releasing calcium in PBS at a physiological pH.

### 2.3. Wettability and Roughness

**Figure 5:**
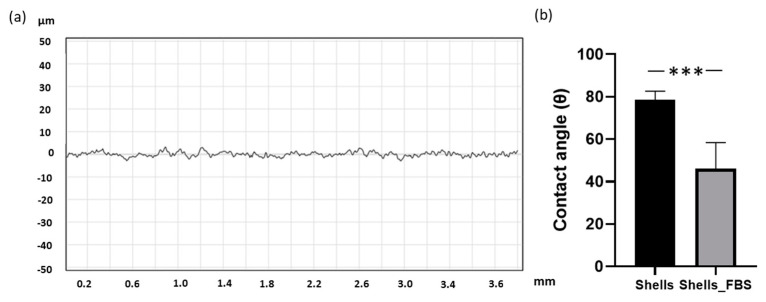
(**a**) Representative profilometry analysis of the mantle surface of *C. fornicata* discs, showing a micro-meter scale roughness. (**b**) Contact angle quantitative analysis of *C. fornicata* discs with and without coating with FBS (*n* = 3). Comparison between groups was assessed by *t*-test (*** *p* = 0.0001).

In our study, *C. fornicata*’s shell discs made from the mantle of the shells showed a roughness average (Ra) of 1.118 ± 7.795 µm (Figure 5a). The material was hydrophilic hydrophilicity, with an average Theta degree of 78.42 (Figure 5b). To enhance its hydrophilicity, we applied a coating of foetal bovine to the shell, resulting in a reduced Theta angle of 46.00.

### 2.4. Bioactivity

**Figure 6:**
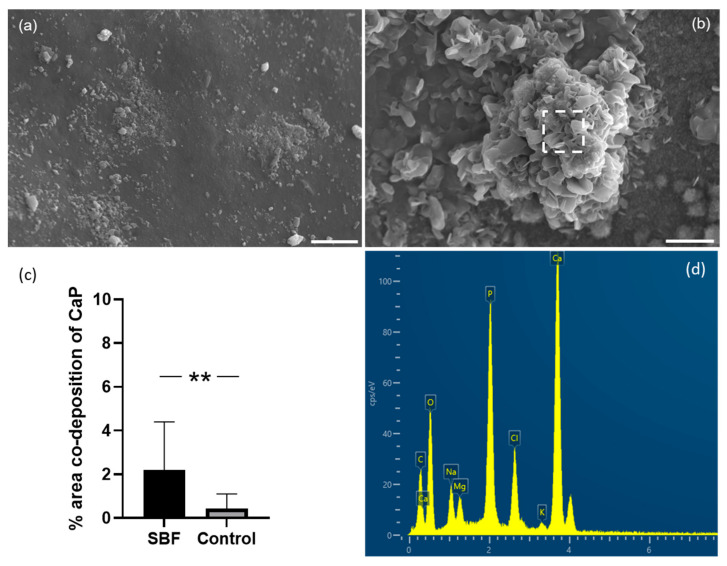
SEM images of *C. fornicata* surface after immersion in either deionized water (**a**) or SBF (**b**) for 21 days. The scale bar is 10 µm. (**c**) Percentage surface co-deposition of calcium and phosphorus as calculated by Imagej from SEM images, showing a significant increase in apatite formation. Results are reported as mean ± SD (*n* = 3). A *t*-test revealed the statistical difference between samples in SBF and controls (** *p* = 0.0026). (**d**). Representative EDS spectrum of the area highlighted by a square in (**b**).

The bioactivity (the ability of a material to form a bond with bone) of a material surface can be assessed by the rate of formation of a crystalline apatite layer, similar to the mineral phase of the bone. Kokubo et al. suggested that apatite formation can be assessed using a solution (a simulated body fluid or SBF) with ion concentrations comparable to human blood plasma [25]. The rate of apatite formation on a surface indicates the level of bioactivity, so surfaces that deposit more apatite are more bioactive. In this study, after a three-week incubation period, discs immersed in SBF showed a significant increase (*p* < 0.01) in Ca and P co-deposition compared to those immersed in water (Figure 6). In addition, the calcium-to-phosphorus ratio was 1.69 ± 0.47, similar to the stoichiometry of human hydroxyapatite [26].

### 2.5. Cell Attachment and Proliferation

**Figure 7:**
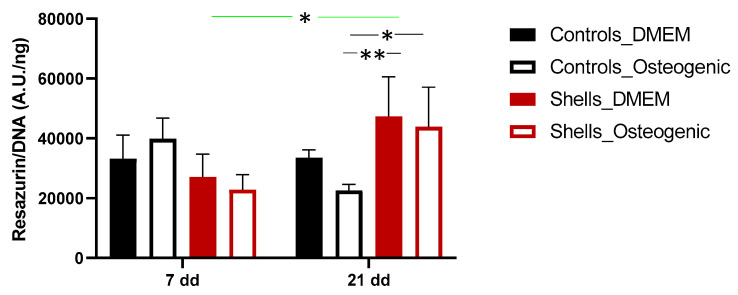
Cell proliferation of hAdMSC to *Crepidula fornicata* shells. Cell proliferation was determined by a resazurin assay. Data are reported as a MEAN ± SD (*n* = 4). Two-way ANOVA returned *p* < 0.05 (* *p* < 0.05 and ** *p* < 0.01).

The ability of the human adipose-derived stem cells to adhere to the disc shells was determined after seeding and growing them in DMEM complete medium, and this was compared to cells that were grown on tissue culture plastic. To induce osteogenic differentiation, the cells were grown in a standard osteogenic medium. In this study, the fluorescence intensity produced by the cells grown on the surfaces was normalized with the DNA content, which reflects the number of cells and more accurately measures cell proliferation rather than just metabolic activity. On day 1, the percentage of cells attaching to the mantle surface coated with FBS was 38.90 ± 17.04%. Figure 7 illustrates the proliferation of cells grown on the surface of the discs for 7 and 21 days. The cells remained viable and proliferated on both tissue plastic surfaces and on the mantle surface. Throughout the experiment, cell proliferation significantly increased for hAdMSC between days 7 and 21, when treated with an osteogenic medium (*p* < 0.01). At day 21, the proliferation of cells on the shell surface was higher than on tissue culture plastic, with a significant increase in the growth of cells on the disc surface when incubated in osteogenic media. The results demonstrate that discs from the mantle coated with FBS support cell adhesion and proliferation.

**Figure 8:**
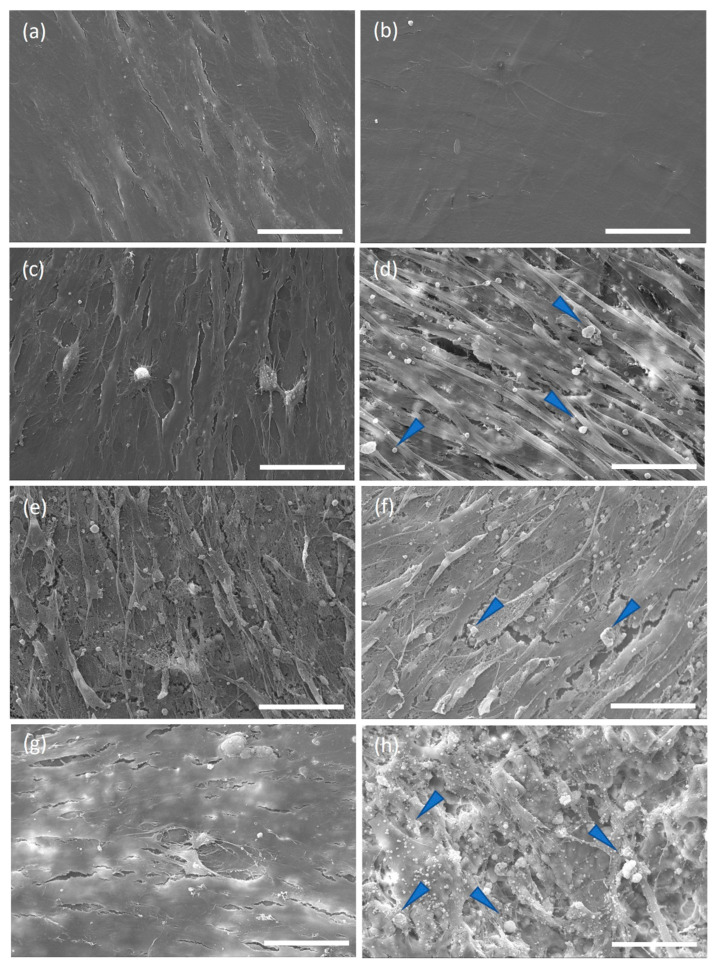
SEM images of hAdMSC grown on coverslips in either DMEM for 7 (**a**) and 21 days (**b**) or osteogenic medium for 7 (**c**) and 21 days (**d**) or on shells with DMEM for 7 (**e**) and 21 days (**f**) or with Osteogenic medium for 7 (**g**) and 21 days (**h**). Images were acquired at 20.00 kV. Arrows indicate mineral deposition on the biomaterial and controls. The scale bar is 50 µm.

To explore the cell morphology when grown on the limpet shells, SEM analyses were performed at 7 and 21 days. Cells grown on coverslips, with complete medium, exhibited a spindle-like shape (typical of confluent stem cells), both at day 7 and 21 (Figure 8). Cells grown on shells formed a multicellular layer covering most of the surface of the discs as early as seven days. Cells grown on the disc surface in the presence of the osteogenic medium were confluent and spindle-like. Notably, hAdMSC grown on both coverslips and the nacre disc surfaces in the osteogenic medium at 21 days showed the formation of nodules, suggesting osteoblastic differentiation and mineral deposition. Whilst nodule formation was seen in the cells grown on the shell surface, it was not seen in cells grown on coverslips in the osteogenic medium at seven days or in cells on coverslips in the non-osteogenic medium at 7 and 21 days. SEM-EDS analyzed the nodules, revealing deposition of calcium and phosphorus minerals, with Ca/P ratios of 0.63 ± 0.30 for cells on coverslips in osteogenic medium, 1.39 ± 0.03 for cells on the shell surface with DMEM and 1.11 ± 0.11 for cells on the shell surface with the osteogenic medium.

### 2.6. Alkaline Phosphatase Activity

**Figure 9:**
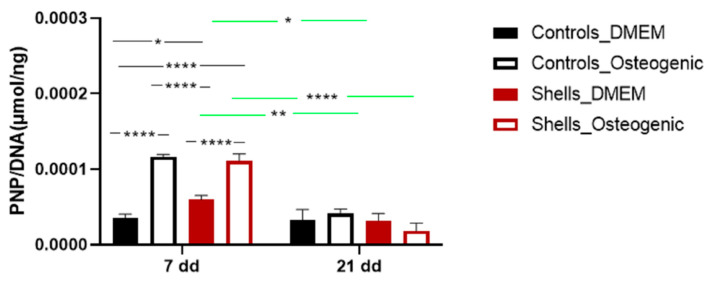
ALP expression of hAdMSC grown on coverslips and shells at days 7 and 21. Data are reported as a MEAN ± SD (*n* = 4). Two-way ANOVA returned *p* < 0.05 (* *p* < 0.05, ** *p* < 0.01 and **** *p* < 0.0001).

Figure 9 shows the ALP expression of hAdMSC grown on coverslips and discs from shells on days 7 and 21. At seven days, there was a significant increase in ALP expression for cells grown on disc compared with those grown on the coverslips. Adding osteogenic supplements results in a higher expression of ALP for cells both on the coverslips and on the disc surface at seven days. There was no difference in ALP expression at this point for cells in osteogenic medium for cells on coverslips compared to those on the shell surface. At 21 days, the levels of ALP expression in cells on both surfaces reduced compared to those at seven days. At this time, the osteogenic medium did not affect the ALP expression of cells on either the coverslip or discs made from the shells.

### 2.7. RT-qPCR

**Figure 10:**
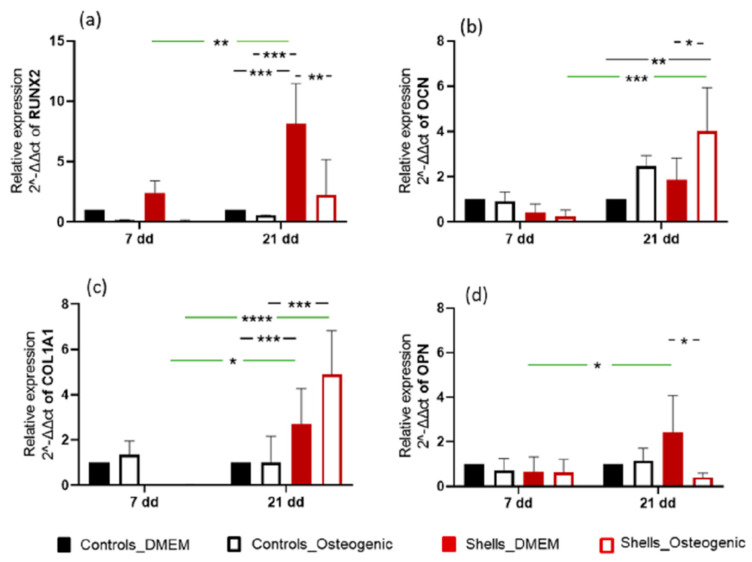
Relative gene expression of RUNX2, COL1, OPN, OCN and Col1A1 of hAdMSC on shells on days 7 and 21. Data are reported as a MEAN ± SD (*n* = 4). Two-way ANOVA returned *p* < 0.05 (* *p* < 0.05, ** *p* < 0.01, ***p < 0.001 and **** *p* < 0.0001).

To further assess the osteogenic differentiation on the surfaces, quantitative real-time PCR measurements were performed on hAdMSC in culture for up to 21 days (Figure 10). The levels of osteogenic markers, namely RUNX2 (runt-related transcription factor 2), OPN (osteopontin), OCN (osteocalcin), and COL1 (collagen type 1 alpha 1 chain, were evaluated. RUNX2 is an early marker of osteogenic differentiation, binding to the promoter regions of genes involved in bone formation. In addition, RUNX2 is also involved in maintaining the activity of osteoblast, as it regulates the expression of genes involved in bone matrix production and mineralization. On day 7, the expression of RUNX2 was upregulated only for hAdMSC grown on discs from shells in basal medium. On day 21, RUNX2 expression was further upregulated in the basal medium—the expression of OPN, a non-collagenous protein that is a late marker of osteogenesis. OPN is normally secreted by osteoblasts, where it interacts with hydroxyapatite, facilitating its deposition onto the bone matrix. Although OPN was upregulated on hAdMSC grown on discs from shells with basal medium, it was not significantly different from the cells on the coverslips. OCN is a non-collagenous late marker of osteogenesis and is secreted by osteoblasts and promotes the deposition of hydroxyapatite. On day 21, osteocalcin (OCN) was upregulated in cells on the disc surface grown in an osteogenic medium, and this was significantly greater compared with cells on coverslips in osteogenic media (*p* < 0.01). Collagen I is not considered a direct marker of osteogenesis, but it is an essential component of the extracellular matrix of bone. It is involved in the mineralization and structure of the bone. Generally, this gene is upregulated during bone formation along with other osteogenic marker genes such as RUNX2, OPN and OCN. COL1A1 was upregulated and statistically different (*p* < 0.001) on day 21 for hAdMSC grown on discs from the shells with basal and osteogenic media. The gene expression results suggest that hAdMSC cultured on discs from shells leads to a stronger osteoinductive response than the cells grown on coverslips, even with the osteogenic medium.

## 3. Discussion

Bone tissue engineering aims to produce biomaterials that can substitute and induce the regeneration of damaged tissue. These materials require either the introduction of cells capable of reconstructing the tissue or the recruitment of such cells from local or systemic sources, such as mesenchymal stem cells and osteoblasts [27,28].

In the current in vitro study, we investigated the physicochemical characteristics of slipper limpet discs and their ability to support the attachment, spreading and osteogenic differentiation of hAdMSCs. This work has sought to identify a novel biomaterial for industrial use with a low ecological and economic impact.

In our study, XRD and FT-IR showed that limpet shells were composed mainly of calcium carbonate in the form of aragonite. These results are consistent with Batzel et al. where XRD using powdered *C. fornicata* shells showed they were made of the calcium carbonate polymorph aragonite with no indication of the presence of other crystalline minerals [29]. In addition, we observed that the surface of the shell naturally exhibited a uniform micro-roughness that was slightly hydrophilic. Hydrophobicity and roughness are crucial surface properties for interaction of a biomaterial with the biological environment, including tissues and cells [24]. Surface topography is known to influence cell adhesion, proliferation, and differentiation. For example, Faia-Torres et al. demonstrated that, on polycaprolactone surfaces, gradients with micrometer-scale roughness supported greater osteogenesis than sub-micrometer surfaces [25]. Similar results were reported by Li et al. on titanium surfaces, where the osteogenesis of human osteoblastic SaOS-2 cells was enhanced with increased surface roughness (from approximately 100 to 400 nm) [30]. However, Davison et al. have shown that in vivo, beta-tricalcium phosphate (TCP) graft substitutes with a submicron-scale surface induced significantly improved bone formation than microstructured TCP [31]. The interfacial free energy of the implant surface is another physicochemical property that influences its interaction with biological environments. Generally, the higher the surface energy, the higher the hydrophilicity, and the higher the cell adhesion. It has been attributed to the rapid spreading of serum on the surface, which provides an overall coating of bioactive factors that can influence early cell adhesion, proliferation, and differentiation [32].

In our work, *C. fornicata* discs, incubated in PBS, could release Ca^2+^ over the experimental period of 7 days. Ca^2+^ in the fluid surrounding cells plays a key role in osteoinduction. It has been shown that the differentiation of human bone marrow-derived MSC towards osteoblasts is accompanied by the expression of Ca^2+^ binding proteins [33].

Osteotransduction, which refers to transforming a biomaterial into new bone tissue after implantation, is a property of calcium phosphate biomaterials. Hydroxyapatite (HAP) formation on the surface is desirable for an implant because HAP can form strong bonds with natural bone. We, therefore, assessed whether discs made from the mantle of *C. fornicata* could promote the formation of calcium phosphate on their surface. We showed that *C. fornicata* discs, immersed in SBF for 21 days, presented Ca/P deposits on their surface [21,34]. In addition, the shell surface was partially covered by spherical nodules composed of calcium phosphate, as shown in EDS. The level of 4% is the area that was associated with both calcium and phosphate. Although the areas occupied by the nodules are not large, the results show that the shell surface still induces osteoinduction.

Increasing the surface area of the aragonite by roughening it or by making it porous would potentially increase calcium release and enhance hydroxyapatite deposition. Interestingly, in our study, the calcium-to-phosphorus ratio was 1.69, which is very similar to the ideal ratio for natural hydroxyapatite, 1.67 [26]. There are two main theories to explain why aragonite, and in particular nacre, can promote hydroxyapatite deposition. One theory is that nacre has an organic component performing a function similar to collagen in bone and promoting hydroxyapatite formation [26]. Alternatively, Ni and Ratner hypothesized that the nacre surface is activated after calcium ions are released from the shells [35]. The free calcium ions bind to phosphate ions and precipitate on the nacre surface as hydroxyapatite. Calcium ions do not re-precipitate as calcium carbonate because the solubility of HAP is much lower than that of aragonite. As seen in our study, when immersed in SBF, calcium ions were released from the *C. fornicata* discs immediately and this continued for seven days, which was the duration of the experiment. Overall, our results suggest that *C. fornicata* discs have the potential to be a valuable biomaterial for bone tissue engineering owing to their ability to release calcium ions and promote the formation of hydroxyapatite on their surface and this promotes osteoblastic differentiation of stem cells.

In this study, human adipose-derived MSCs were used to investigate osteogenesis, as they have the potential to differentiate into osteoblasts [36]. The fluorescence quantification of resazurin and the SEM images demonstrated that the cells could attach and proliferate on discs from shells, as previously described for hAdMSC grown on other shell surfaces [21]. Interestingly, despite the cell attachment efficiency of 38% after one day on shell surfaces compared to the coverslips, by day 7, the proliferation was similar. This suggests that hAdMSC proliferated faster on *C. fornicata* than on the coverslip surface. Similar observations were made by Kamba and Zakaria using hFOB 1.19 co-cultured with calcium carbonate nanocrystals derived from cockle shells [37]. In our study, cells grown on both materials presented a spindle-like shape on days 7 and 21. Furthermore, the mineral deposition and the extracellular matrix production by cells on the shells in a non-osteogenic medium indicate that the shells promoted osteoblastic differentiation.

We quantified the relative gene expression of early (RUNX2 and Col1A1) and late bone-specific markers (OPN and OCN) to confirm osteogenic differentiation. These early and late bone-specific markers have been well-documented in previous reports [38]. The cells used for this study were a multipotent human adipose-derived stem cell line, which can differentiate into different cell lineages, such as adipocytes, myocytes, chondrocytes, and osteoblasts. In vivo, differentiation depends on the composition and 3D organisation of the extracellular matrix (ECM) and the combined effect of growth factors and other morphogens in their immediate environment [39]. In our in vitro study, osteogenic differentiation was initiated by the shell. RUNX2 is essential for osteogenic differentiation and regulates the temporal expression of other osteogenic markers and the secretion of the extracellular matrix. COL1A1 expression was higher at 21 days than at seven days, for the cells grown on *C. fornicata* shells, both with and without an osteogenic medium. Osteocalcin expression was significantly greater when cells were grown on *C. fornicata* shells indicating that the shells promoted gene expression related to osteogenic differentiation. RUNX2 upregulation was not recorded for cells grown on *C. fornicata* in complete medium but was when the osteogenic medium was used. Nevertheless, the effect of growing cells on the shell surface was to promote mineralization even when incubated in DMEM without an osteogenic medium.

To evaluate markers of osteogenesis, we also examined the activity of the alkaline phosphatase (ALP), an early and transient indicator of osteogenic differentiation. ALP was present in all cells on day 7. The ALP activity was significantly higher for cells grown on shells without an osteogenic medium. Adding osteogenic medium at day 7 increased the levels of ALP for cells on coverslips and on shells suggesting that the osteogenic media suppressed the surface effect. Corrêa de Almeidaa et al., working with *Limnoperna fortunei* shells (also predominantly aragonite), didn’t find any increase of ALP activity in human adipose stem cells grown for five days in DMEM medium, suggesting that *C. fornicata* shells may be better promoters of bone formation [21]. However, when Corrêa de Almeidaa et al. powdered the same shells, there was a significant increase in ALP activity due to the larger contact surface, which considerably increased calcium release in the media, favoring a faster osteogenic process. In our study, ALP activity diminished for all groups on day 14 except for the control group. The decline of ALP activity might be associated with the rapid formation of minerals. ALP is required before the onset of matrix mineralization, providing localized enrichment of inorganic phosphate, one of the components of apatite [40]. In our study, SEM-EDS images showed mineralization at day 7, indicating that earlier time points for ALP measurement may have shown an even larger response to the shell surface.

The results suggest that the shell composition is partially responsible for the mineralization of the extracellular matrix, as shown by the differentiation of the stem cells and the hydroxyapatite deposition. Furthermore, our results indicate that *C. fornicata* shells (regardless of using osteogenic medium) promote bone formation. Although the shell material would need to be fabricated into a bone graft substitute material which would depend on the application, its availability and relatively low cost make it an attractive option with a low carbon footprint. Future studies should focus on using granular material derived from shells and possibly developing a porous scaffold made from shells to mimic bone’s porous interconnected network which facilitates vascularization and rapid growth of newly formed bone.

## 4. Materials and Methods

### 4.1. Preparation of Samples

**Figure 11:**
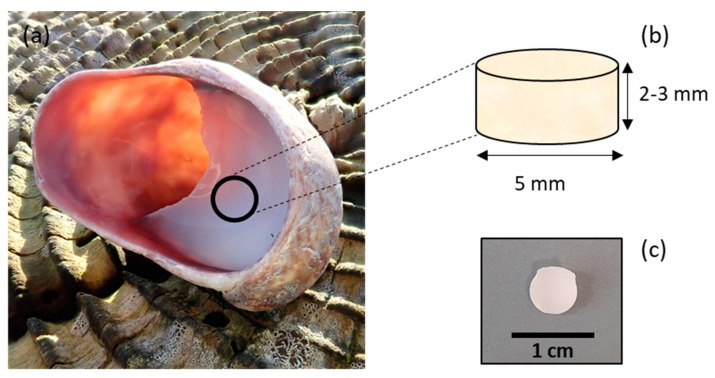
Fabrication procedure of *C. fornicata* discs. (**a**) Cylinders were shaped by coring the mantle of rinsed slipper limpet shells using a diamond core. (**b**) Cylinders had an average diameter of 5 mm and an average height of 2–3 mm. (**c**) Representative *C. fornicata* disc.

*C. fornicata* shells were collected in Pembrokeshire, Wales, UK. The samples were rinsed with distilled water. The samples were then air-dried at room temperature. Shells were shaped as cylinders by coring the mantle of the shell with a 5 mm diameter diamond core fitted into a pillar drill (Sealey, Suffolk, UK) (Figure 11).

### 4.2. FT-IR

*C. fornicata* powder was obtained by grinding the shells to a fine powder in a ceramic mortar. FT-IR spectra of *C. fornicata* powder were recorded using a Varian FT-IR 640-IR Instrument (Agilent, Santa Clara, CA, USA) and spectra were processed using Agilent Resolution Pro software (Agilent Technologies, Mulgrave, Australia).

### 4.3. XRD

*C. fornicata* powder was prepared as described above. X-ray diffraction was performed using a X’ pert3 powder X-ray diffractometer (Malvern PANalytical B.V., Almelo, The Netherlands). The dataset was collected using Cu Ka radiation in the 2θ range of 5 to 89 at a scan speed of 0.006°2θ/min. The generator settings were 35 mA and 40 kV.

### 4.4. SEM and EDS

Scanning electron microscopy of shells coated with gold/palladium (Au:Pd 80:20) using a Quorum 150 TES sputter coater was undertaken by a Tescan MAIA 3 scanning electron microscope (15 kV). For SEM-EDS analysis, the specimens were coated with an automatic SEM carbon coater (Agar Scientific, Stansted, UK). Then the samples were imaged by an EVO MA10 SEM (Carl Zeiss, Cambridge, UK) fitted with a LaB_6_ electron source. Elemental composition was determined by X-ray spectroscopy maps (20 kV) collected using an Oxford Instrument X-Max 80 detector.

### 4.5. Calcium Release Studies

The discs were rinsed with water, autoclaved, and finally soaked in 1 mL of PBS (without calcium and magnesium) (Fisher, Loughborough, UK) in sterile tubes. The samples were incubated at 37 °C with shaking (100 rpm). At scheduled time points (0.5, 1, 2, 4, 24, 28, 72 and 168 h), 0.5 mL of solution was taken and replaced with the same volume of fresh PBS. The concentration of calcium was determined with a calcium colorimetric kit (Merck Life Science, Gillingham, UK) according to the manufacturer’s instructions. Briefly, 50 μL of samples were mixed with 90 μL of chromogenic reagent and 60 μL of calcium assay buffer. Then, the plates were incubated at room temperature for 10 min, and the absorbance was read at 575 nm (SpectraMax i3x) (Molecular Devices, San Jose, CA, USA), using PBS as a blank. The experiment was conducted in triplicate.

### 4.6. Roughness Analysis

The surface roughness of the *C. fornicata* discs was measured using a Formtracer SV-C3200 instrument and analysed using the FORMTRACEPAK software (Mitutoyo UK Ltd., Andover, UK). The samples were kept steady using blue tack. The measured length was 4 mm, the measuring speed was 1.00 mm/s, the measuring pitch was 0.0005 mm, and the Z1-axis range was 800 μm. The measurements were repeated three times per sample, and the experiment was done in triplicate.

### 4.7. Wettability

Contact angle measurements for each FBS-coated (made as described in paragraph 4.9) and uncoated disc were performed using an optical contact angle meter CAM101 (KSV Instruments Ltd., Espoo, Finland). The measurements were conducted with deionized water and were repeated three times for each surface.

### 4.8. In Vitro Bioactivity

The deposition of apatite on the surface of *C. fornicata* from simulated body fluid was assessed according to the methods of Kokubo and Takamada [20]. Sodium chloride (NaCl), sodium hydrogen carbonate (NaHCO_3_), potassium chloride (KCl), di-potassium hydrogen phosphate trihydrate (K_2_HPO_4_.3H_2_O), magnesium chloride (MgCl_2_), calcium chloride (CaCl_2_), sodium sulfate (Na_2_SO_4_), tris-hydroxymethyl aminomethane ((HOCH_2_)_3_CNH_2_) and hydrochloric acid were bought from Fisher (Loughborough, UK). The autoclaved samples were soaked in 15 mL of SBF in 50 mL tubes. Samples were incubated at 37 °C for 21 days. Samples kept in deionized water were used as a control. The SBF was changed every 3–4 days. Finally, the samples were gently washed in deionized water and vacuum dried. Samples were carbon-coated and imaged as described above. EDS images for calcium (Ca) and phosphorus (P) were analyzed using ImageJ (NIH, Madison, WI, USA) after being overlaid to corresponding SEM images. The total area of overlapping for Ca and P onto the total sample surface area was used to calculate the percentage of Ca and P salt deposition (*n* = 3).

### 4.9. In Vitro Cell Culture

An adipose-derived stem cell line (Asc52telo, ATCC^®^ SCRC-4000^™^) was bought from LGC standards (Teddington, UK). The cells tested negative for HIV, HepB, HPV, EBV, CMV and mycoplasma. Cells were maintained, under aseptic conditions, in DMEM (high glucose, GlutaMAX™, pyruvate) supplemented with 10% heat-inactivated FBS and 1% penicillin-streptomycin (Fisher, Loughborough, UK). The cells were cultured at 37 °C in a 5% CO_2_ humidified incubator, and the culture medium was changed twice weekly. At 80–90% confluence, the culture medium was removed, and the cells were washed with Dulbecco’s phosphate-buffered saline (DPBS) (Fisher, Loughborough, UK). The cells were detached following the incubation with 0.25% trypsin-EDTA (Fisher, Loughborough, UK) at 37 °C. Cells between passages 4 and 5 were used for the study.

### 4.10. Cell Seeding on Discs

Discs were autoclaved and then put in 48 (low cell attachment) well plates. Discs were coated with FBS for 4 h and air-dried, under laminar flow, overnight. Human adipose-derived stem cells (60,000) were seeded in 20 μL of complete medium were seeded on each sample and control (12 well plates). More precisely, 30,000 cells in 10 μL were seeded on one side of the disc and then incubated for 90 min. The same was repeated for the opposite side of the scaffold. Then, the samples were covered with a complete medium and incubated (5% CO_2_ and 37 °C). After one day, the discs were transferred to a new plate, covered with either a basic or osteogenic medium (StemPro^TM^ Osteogenesis differentiation kit) (Fisher, Loughborough, UK), and cultured for 21 days.

### 4.11. Cell Attachment and Proliferation

After 24 h since seeding, a resazurin assay (Merck, Gillingham, UK) was performed. A filter sterile 0.1 mM resazurin salt solution in a complete medium was added to each sample. The plates were incubated (5% CO_2_ and 37 °C) for 2 h. The working solution, incubated in the same conditions, worked as a blank. Then, 100 µL aliquots were transferred to a new 96-well plate, and the fluorescence was immediately read using a plate reader (ex/em 540/585 nm). The experiment was performed in triplicate (*n* = 3). At the end of the experiment, the resazurin solution was removed, and a fresh medium was added.

### 4.12. Alkaline Phosphatase Activity (ALP)

Carbonate lysis buffer (450 μL) (1% Triton in 0.2 M carbonate buffer, pH 10) (Merck, Gillingham, UK) was added to the shells and controls. Afterwards, the extracts were frozen/thawed thrice and lysed using a 21 G needle. For ALP quantification, 50 μL of lysate was mixed with 100 μL of p-Nitrophenyl Phosphate (pNPP, 6 mM) carbonate solution (containing 10 mM MgCl_2_) (Merck, Gillingham, UK). The plate was incubated at 25 °C for 1 h. Absorbance was read at 405 nm. Data were normalised against DNA. DNA quantification was done using ab156902 General DNA Quantification Kit (SYBR Green) (Abcam, Cambridge, UK), according to the manufacturer’s instructions. More precisely, 5 µL of extracts were mixed with 100 µL of 1X DNA assay solution and the fluorescence was read, after 10 min, (480/520 ex/em. The specific activity of cells was expressed as μmoles/ng DNA (±SD).

### 4.13. Cell Morphology by SEM

On days 7 and 21, samples were fixed in 2.5% glutaraldehyde (Merck, Gillingham, UK) in 0.1 M cacodylate (Merck, Gillingham, UK) overnight at 4 °C. The samples were then washed with deionized water three times and dehydrated with an ethanol series (20 min each, 50%, 70%, 90% and 100%) (Fisher, Loughborough, UK) and with 1:2 and 2:1 HMDS (hexamethyldisilazane): EtOH and 100% HMDS (overnight) (Merck, Gillingham, UK). The samples were then mounted on stubs and gold-coated (Polaron e500) (Quoram Technologies, Laughton, UK) before imaging at 20.00 kV by SEM.

### 4.14. RNA Extraction and RT-qPCR

Qiazol lysis reagent (Qiagen, Manchester, UK) was used to lyse cells at 7 and 21 days. Cell content was homogenised using a sterile 25-gauge needle and centrifuged at 18,000× *g* for 2 min at room temperature. The supernatant was processed using a RNeasy PLUS micro kit for RNA isolation (Qiagen, Manchester, UK). RNA was quantified using a nanodrop (Nanodrop ND-1000) (Thermo Fisher Scientific, Wilmington, NC, USA) and stored at −80 °C until use. Complementary DNA (cDNA) was obtained from RNA (250–500 ng from each sample) using High-Capacity cDNA Reverse Transcription Kit (Fisher, Loughborough, UK) on a thermal cycler (T100) (Biorad, Watford, UK), according to the manufacturer’s instructions. RTq-PCR was performed in a LightCycler96 (Roche, Burgess Hill, UK), using SSO Universal SYBR Green Supermix (Fisher, Loughborough, UK), and data were analysed using the LightCycler SW 1.1 analysis software (Roche, Penzberg, Germany). Relative gene expression was calculated using the comparative 2^−ΔΔCt^ (expressed as a fold change). GAPDH was used as a housekeeping gene. The primers used were GAPDH (housekeeping gene), RUNX2, OPN, OCN and COL1A1 (Eurofins Genomics, Ebersberg, Germany) (Table 1).

### 4.15. Statistical Analysis

Data was parametric, so statistical significance was analysed using the unpaired *t*-test and ANOVA and all results are shown as mean ± SD. Samples were run in triplicate for the biochemical assays. Statistical analysis was performed using GraphPad Instant Software (GraphPad Software Inc., Boston, MA, USA). All experiments were repeated at least three times.

## 5. Conclusions

This study shows that *C. fornicata* discs exhibited bioactivity and biocompatibility with osteogenic potential. This study suggests that the material might be a promising alternative to coralline aragonite and calcium phosphate ceramics. Should a hydroxyapatite surface be more suitable for bone formation, the calcium carbonate at the shell surface could be converted using a hydrothermal process [41]

Future studies should focus on using fragmented shells to evaluate whether the increase in the contact surface would improve osteogenic performance. The fragmented structure may better mimic the porous interconnected network to facilitate vascularization and rapid growth of newly formed bone. It would also be valuable to investigate in vitro and in vivo resorption of the surface by dissolution or by an osteoclastic or macrophage interaction. Ultimately, before this material could be used in humans, an in vivo study to examine the effect at the tissue level and the interaction with the immune system, blood vessels, and other bone cells is a prerequisite.

## Figures and Tables

**Table 1:** Primers for qPCR.

Gene Name	Forward	Reverse
GAPDH	CCGCATCTTCTTTGCGTCG	GCCCAATACGACAAATCCGT
RUNX2	CCACCGAGACACCATGGAG	CGCCTGGGTCTCTTCACTAC
OPN	TAGGCATCACTGTGCCATAC	TACTGGAAGGGTCTGTGGGG
OCN	CACTCCTCGCCCTATTGGC	CCCTCCTGCTTGGACACAAAG
COL1A1	TGGCAAGAACGGGATGACG	GCACCATCCAAACCATGAA

GAPDH Glyceraldehyde 3-phosphate dehydrogenase, RUNX2 Runt-related transcription factor 2, OPN Osteopontin, OCN Osteocalcin and COL1A1 Collagen type 1 alpha 1 chain.

## Data Availability

The data presented in this study are available on request from the corresponding author.
